# Origin of ventricular fibrillation triggers in a model of localized repolarization heterogeneity

**DOI:** 10.1016/j.hrthm.2024.10.027

**Published:** 2025-05

**Authors:** Estelle Renard, Michel Haïssaguerre, Laura R. Bear, Olivier Bernus

**Affiliations:** 1 Univ. Bordeaux, INSERM, CRCTB, U 1045, IHU Liryc, Bordeaux, France; 2 CHU de Bordeaux, Service de Cardiologie-électrophysiologie et stimulation cardiaque Hôpital Haut Lévêque, INSERM, U 1045, Bordeaux, France

**Keywords:** Sudden cardiac death, Ventricular fibrillation, Repolarization, Dispersion, Premature ventricular complex, Trigger, Substrate

## Abstract

**Background:**

Heterogeneities in ventricular repolarization contribute significantly to the genesis of ventricular fibrillation (VF). Although clinical arrhythmias are spontaneously triggered by premature ventricular complexes, these triggers are difficult to document and little is known about their site of origin.

**Objectives:**

The purpose of this study was to characterize spontaneous VF initiation in an experimental model of repolarization heterogeneity and to identify the origin of triggers in relation to the spatial dispersion of repolarization.

**Methods:**

Spatially limited repolarization heterogeneity was created in isolated perfused porcine right ventricles (N = 16) by local administration of pinacidil (20 μM) in a terminal branch of the right coronary artery. High-resolution optical mapping and pseudo-electrocardiography were performed under control conditions and after pinacidil perfusion.

**Results:**

No arrhythmia occurred at baseline, but 74 VF episodes were observed in 13 hearts (82%) after pinacidil perfusion and were most often initiated by a ventricular trigger with a short coupling interval (297 ± 66 ms). Sixteen VF initiations were optically mapped in 4 hearts. Mapping showed triggers originating in all cases from the border zone between altered and normal repolarization areas where local action potential duration and repolarization time gradients were steep (15.9 and 15.8 ms/mm vs 1.5 and 3.0 ms/mm at nontrigger sites). Optical action potential traces were compatible with a phase 2 reexcitation mechanism. The subsequent VF cycles were driven by activities located in the same region.

**Conclusion:**

This model of localized repolarization heterogeneity is able to produce spontaneous VF initiation. Our study demonstrates that VF triggers originate consistently from the border zone of repolarization dispersion.

## Graphical abstract

**Figure:**
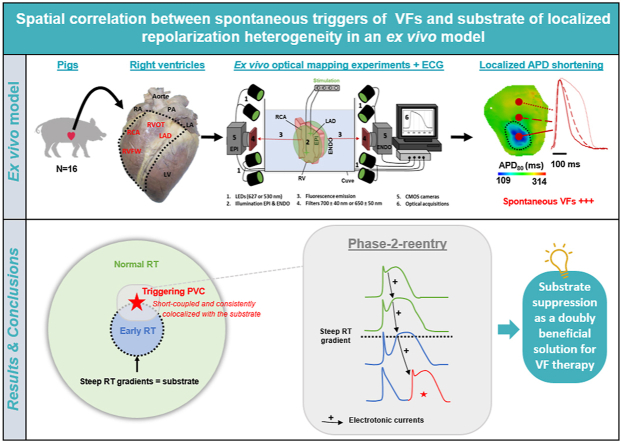

## Introduction

Ventricular arrhythmias and sudden cardiac death are a major public health issue responsible for approximately 10% of deaths in human adults.^1^, ^2^, ^3^ They are mostly associated with preexisting heart disease but can also occur in the absence of a manifest structural cause.^4^, ^5^, ^6^, ^7^, ^8^, ^9^, ^10^, ^11^ Abnormal repolarization syndromes (long QT, short QT, Brugada, and early repolarization syndromes) are cardiac disorders frequently responsible for ventricular fibrillation (VF) in the young. Clinical studies have shown that VFs are often triggered by premature ventricular complexes (PVCs) originating from Purkinje or myocardial tissue and occurring early and superimposed on the preceding T wave.^5^^,^^12^, ^13^, ^14^ The major role of triggers is demonstrated by localized ablation resulting in the elimination or decrease of arrhythmic recurrences.^13^, ^14^, ^15^, ^16^, ^17^, ^18^, ^19^ The genetic and cellular mechanisms of the substrates underlying repolarization syndromes have been considerably clarified in the last decades, and the role of dispersion of repolarization time (RT), with or without structural alteration, has been described as a critical determinant of arrhythmogenesis.^20^, ^21^, ^22^, ^23^, ^24^ However, few studies have focused on *spontaneous* VF initiation at the organ level, in contrast to pacing-induced VF, and the association between VF triggering and underlying electrical abnormalities.

The aims of our study were to identify the site of origin of spontaneous VF triggers by using high-resolution optical mapping in a pharmacologically induced repolarization heterogeneity ex vivo model and to assess its specificities compared to other nonarrhythmogenic sites.

## Methods

**Figure S1:**
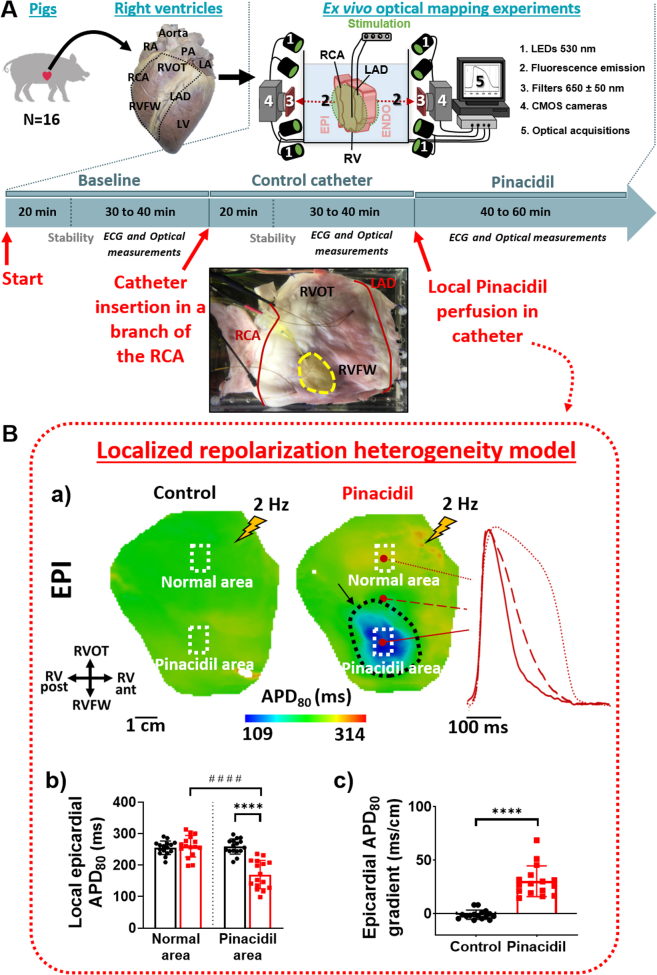


Further methodological details are provided in the Online Supplement, with the workflow of the experiments summarized in Online Supplemental Figure 1.

### Animals

The experiments were performed using 16 isolated dual-perfused right ventricles from large white male pigs aged 3 months ± 2 weeks and weighing 40.1 ± 1.9 kg. All the protocols were in accordance with the European Parliament Directive 2010/63/EU and approved by the local ethics committee (CEEA-050) and the national regulations (approval no. A33-318-3).

### Model of localized repolarization heterogeneity

Our model of localized repolarization heterogeneity has been described previously.^25^ After the measurements under control conditions, a catheter was inserted into a distal branch of the right coronary artery. The adenosine triphosphate-sensitive potassium channels opener, pinacidil (20 μM), was then infused through this catheter to create a regional shortening of local action potential duration (APD) (−89.0 ± 48.4 ms in the pinacidil area ≈ 5%–10% of the ventricular tissue ≈ 1–2 cm^2^), which was surrounded by normal areas, thus creating localized repolarization heterogeneity.

### Optical mapping and pseudo-electrocardiographic measurements

Pseudo-electrocardiograms (ECGs) were recorded to allow continuous monitoring of global electrical activity and reanalyze retrospectively all experiments to quantify spontaneous arrhythmias (LabChart acquisition system and software, AD Instruments, Oxford, United Kingdom). Optical mapping was performed continuously for 30–60 minutes (in contrast to usual short acquisitions lasting up to 8 seconds) to capture spontaneous VF initiation if it occurred. VFs were considered spontaneous if they occurred when the preparation was not paced (ie, it had its own intrinsic/automatic rhythm) or in case of pacing when it occurred independently of any extrastimulation or rapid pacing (>2 Hz) protocol. VF episodes were classified as either sustained (≥8 seconds) or nonsustained (<8 seconds). VF initial cycles were mapped using activation and phase mapping to identify the spatial origin of activities (acquisition systems: MiCAM02-CMOS in N = 13 and then MiCAM03-N256 in N = 3; BV Workbench software, SciMedia Ltd, Brain Vision, Costa Mesa, CA).

### Statistical analysis

Statistical tests were conducted using Prism 10.3.1 (GraphPad Software). Continuous variables are expressed as mean ± standard deviation. The comparison of 2-paired or unpaired variables was performed using the appropriate Student statistical test (paired *t* test or unpaired *t* test) or its nonparametric equivalent (Wilcoxon signed rank test or Wilcoxon rank sum test, respectively) when the populations did not have a normal distribution (tested by Shapiro-Wilk). The comparison of multiple variables was performed using the nonparametric equivalent of the ordinary 1-way (unpaired) analysis of variance (Kruskal-Wallis test) implemented using Holm-Sidak multiple comparison tests. Fisher exact tests were used to test the independence of 2 categorical variables when required. A *P* value less than .05 was considered statistically significant.

## Results

### Incidence of spontaneous VF

**Figure 1:**
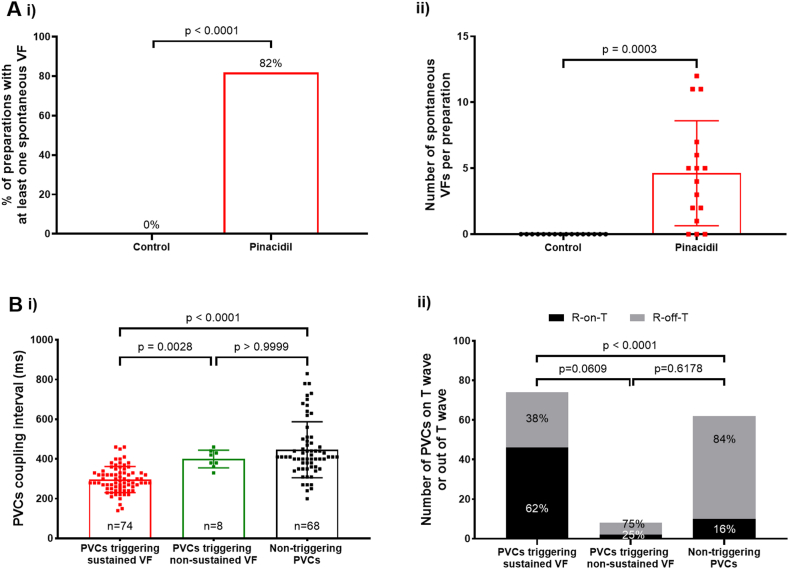
Incidence of ventricular fibrillation (VF) episodes and temporal characteristics of the triggers. **A:** The induced localized repolarization heterogeneity (pinacidil) promoted the appearance of spontaneous VF (no stimulation or constant pacing <2 Hz). (**i**) Percentage of preparations that presented at least 1 spontaneous VF (Fisher exact test; N = 16). (**ii**) Number of spontaneous VF episodes per preparation (data are presented as mean ± standard deviation (SD); paired *t* test; N = 16). **B:** Comparison of the temporal characteristics of premature ventricular complexes (PVCs) triggering sustained VF with PVCs triggering nonsustained VFs and nontriggering PVCs. (**i**) Coupling interval (data are presented as mean ± SD; nonparametric equivalent of the ordinary 1-way analysis of variance, that is, Kruskal-Wallis test implemented using the Holm-Sidak multiple comparison test; n = 74, n = 8, and n = 68; N = 16). (**ii**) T-wave temporal correlation (Fisher exact test; n = 74, n = 8, and n = 68; N = 16).

**Figure 2:**
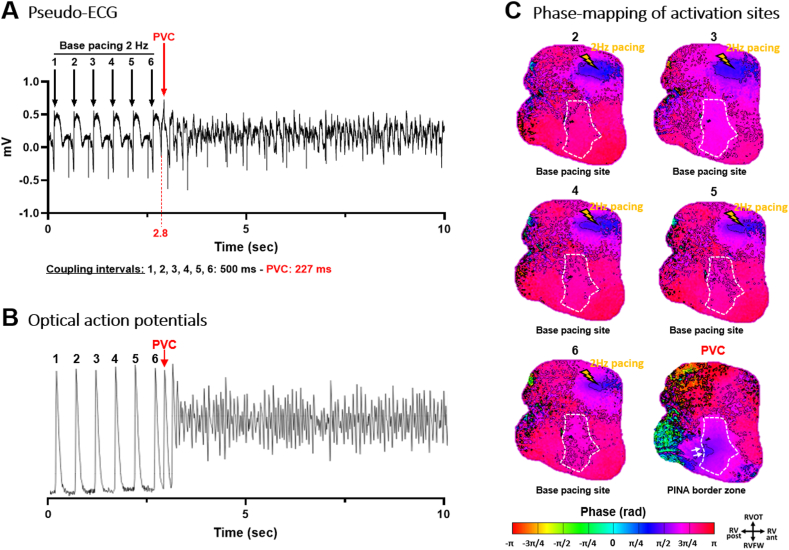
Spontaneous ventricular fibrillation (VF) onset during a constant pacing at 2 Hz in the right ventricular (RV) base. **A:** Pseudo-electrocardiograms (ECGs) of cardiac electrical activities/complexes with a trigger (premature ventricular complex [PVC]) coupling interval of 227 ms. **B:** Corresponding optical action potentials from the pinacidil-perfused area (*white dotted line* in panel C). **C:** Snapshots of the phase mapping movie showing in *blue* the origin of activations of paced electrical cycles 2–6 at the RV base (*yellow arrow*) prior to the start of VF. The last snapshot shows the triggering complex originating from the (*blueareas* – *white arrows*) border zone of the pinacidil-perfused short repolarization area (ie, overlapping short and normal repolarization). PINA = pinacidil; RV ant. = anterior right ventricle; RV post. = posterior right ventricle; RVFW = right ventricular free-wall; RVOT = right ventricular outflow tract.

**Figure 3:**
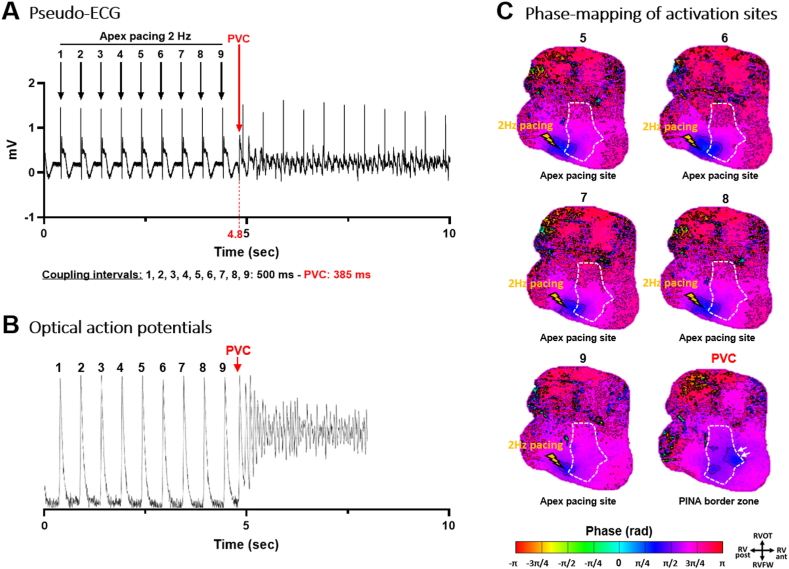
Spontaneous ventricular fibrillation (VF) onset during a constant pacing at 2 Hz in the right ventricular (RV) apex. **A:** Pseudo-electrocardiograms (ECGs) of electrical complexes with a trigger (premature ventricular complex [PVC]) coupling interval of 385 ms. **B:** Corresponding optical action potentials from the pinacidil-perfused area (*white dotted line* in panel C). **C:** Snapshots of the phase mapping movie showing in *blue* the origin of activations of paced electrical cycles 5–9 at the RV apex (*yellow arrow*) prior to the start of VF. The last snapshot shows the triggering complex originating from the (*blueareas* – *white arrows*) border zone of the pinacidil-perfused short repolarization area (ie, overlapping short and normal repolarization). PINA = pinacidil; RV ant. = anterior right ventricle; RV post. = posterior right ventricle; RVFW = right ventricular free-wall; RVOT = right ventricular outflow tract.

**Figure 4:**
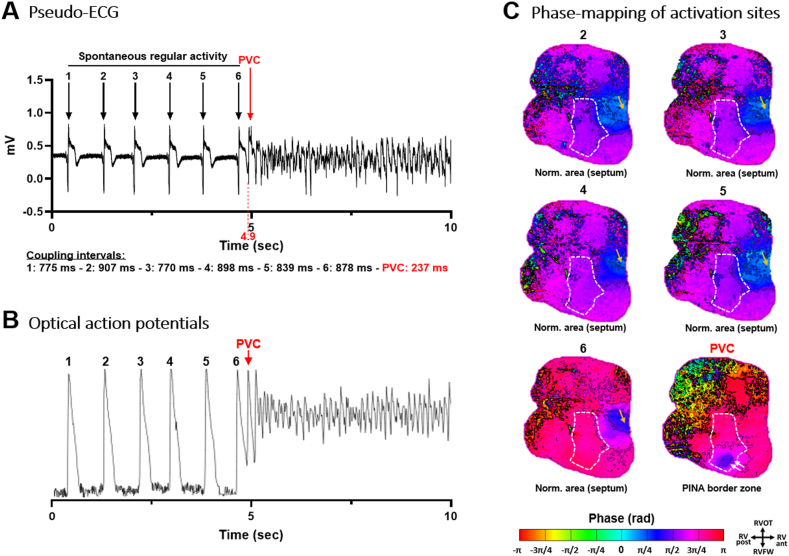
Spontaneous ventricular fibrillation (VF) onset occurring during spontaneous ventricular rhythm in the absence of pacing. **A:** Pseudo-electrocardiograms (ECGs) of cardiac electrical activities/complexes with a trigger (premature ventricular complex [PVC]) coupling interval of 227 ms. **B:** Corresponding optical action potentials from the pinacidil-perfused area (*white dotted line* in panel C). **C:** Snapshots of the phase mapping movie showing in *blue* the origin of activations of spontaneous activities 2–6 from a normal area (interventricular septum) at approximately 1.5 Hz (*yellow arrow*) prior to the start of VF. The last snapshot shows the triggering complex (*blue areas* – *white arrows*) originating from the border zone of the pinacidil-perfused short repolarization area (ie, overlapping short and normal repolarization). PINA = pinacidil; RV ant. = anterior right ventricle; RV post. = posterior right ventricle; RVFW = right ventricular free-wall; RVOT = right ventricular outflow tract.

**Figure S2:**
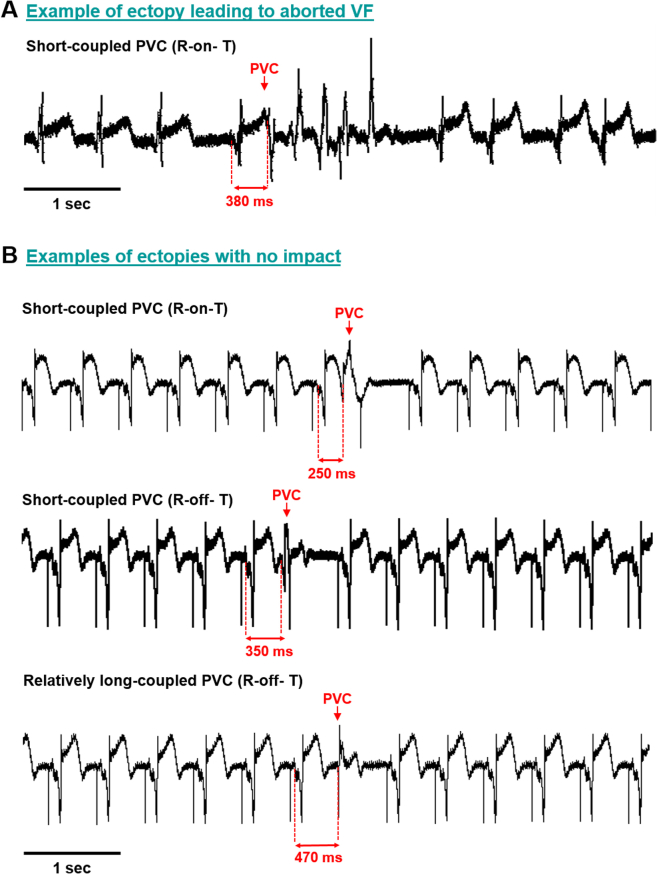


No VF was observed under control conditions, while 13 of 16 preparations (82%) presented at least 1 spontaneous VF after pinacidil perfusion (*P* < .0001). A total of 74 spontaneous VF episodes were recorded using pseudo-ECGs, with an average of 5 per preparation (Figure 1A). Multiple VF episodes in the same preparation showed that VF could be triggered under various conditions, whether pacing from different ventricular sites (examples in Figures 2A and 3A) or during intrinsic activity (example in Figure 4A). The trigger mean coupling interval for all VF episodes was 297 ± 66 ms, with a range of 140–460 ms. The coupling interval of VF triggers was shorter than that of isolated PVCs not inducing VF (447 ± 141 ms; *P* < .0001). Eight episodes of nonsustained VF were observed, which were initiated by triggers with intermediate coupling intervals (400 ± 44 ms) (Figure 1B and Online Supplemental Figure 2). Around 38% of PVCs triggering sustained VFs occurred outside the electrocardiographic T wave, which is lower than in the case of PVCs not inducing VF and PVCs triggering nonsustained VFs (Figure 1B).

### Spatial origin of VF triggers

**Figure 5:**
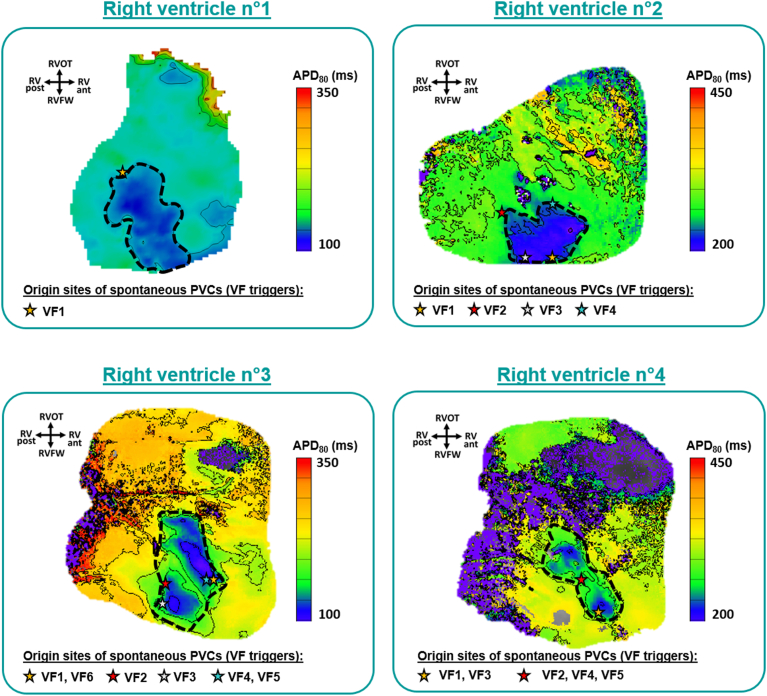
Spatial correlation between the spontaneous ventricular fibrillation (VF) trigger and the pinacidil-perfused short repolarization substrate. The sites of origin of VF triggers (n = 16) are indicated by *stars* superimposed on the corresponding action potential duration (APD) maps (made before the VF onset) of the 4 right ventricular preparations. Each *star* of a different color represent 1 or more VF events (see the legend). The *black dotted lines* outline the pinacidil-perfused area (junction of early repolarization and longer repolarization or “border zone”). All VF triggers were located near the border zone. Note that the *purple pixels* are “noisy” points whose APD could not be calculated. APD_80_ = action potential duration at 80% of repolarization; PVC = premature ventricular complex. PINA = pinacidil; RV ant. = anterior right ventricle; RV post. = posterior right ventricle; RVFW = right ventricular free-wall; RVOT = right ventricular outflow tract.

Sixteen VF initiations were optically mapped (n = 16) in 4 right ventricular preparations (N = 4). Optical maps demonstrated that all the VF triggers originated near the “border zone” (distance of ≤3.5 mm), that is, the junction of early and normal repolarization corresponding to the outline of the pinacidil-perfused area (white and black dotted lines in Figure 2, Figure 3, Figure 4, Figure 5). This result was independent of the rhythm (spontaneous or paced from different sites). Examples of distinct rhythm conditions (those of Figure 2, Figure 3, Figure 4) and all the other cases are shown in Figure 5, demonstrating the clustering of VF triggers, represented by stars, at the border zone of short repolarization areas (in blue). Each star of a different color represents 1 or more triggering PVCs, when some had common origin sites (see the legend on the figure).

**Figure 6:**
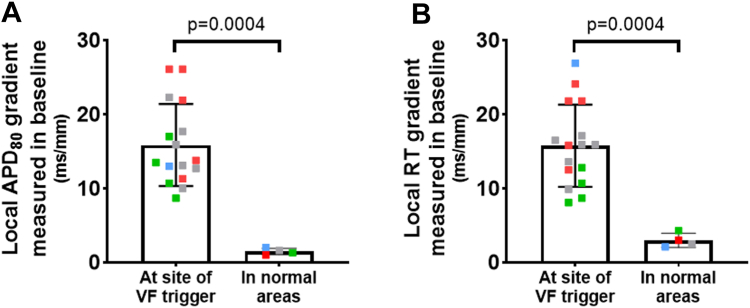
Spontaneous ventricular fibrillation (VF) triggers originated from sites with steep local action potential duration (APD) and repolarization time (RT) gradients. Local (**A**) action potential duration at 80% of repolarization (APD_80_) and (**B**) RT gradients measured during regular activity preceding the onset of VF at the site of VF trigger origin. Each *point* represents 1 optically recorded VF initiation (n = 16), and each color represents 1 right ventricular preparation (N = 4). APD_80_ and RT gradients were compared with those at nontrigger sites (average of 3 pixel measurements taken in the nonaffected sites/normal areas for each preparation; N = 4). Local APD_80_ and RT gradients are significantly steeper than those found in normal areas. Data are presented as mean ± standard deviation; nonparametric unpaired *t* test (Wilcoxon rank sum test).

**Figure 7:**
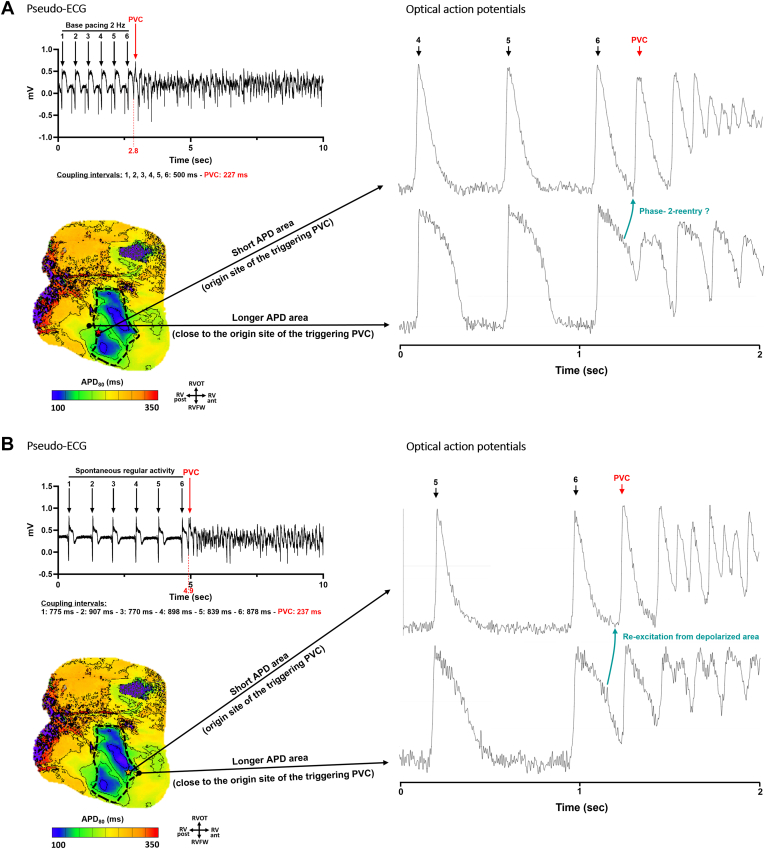
Ventricular fibrillation (VF) trigger potentially caused by phase 2 reentry. Examples of optical action potentials during VF initiation in the short action potential duration (APD) area (early repolarization time [RT]) at the site of trigger origin of VF (*top*), compared with traces from a neighboring area (distance of 5 mm) of longer APDs (*bottom*). The neighboring area is not yet repolarized when the PVC trigger arises, suggesting the possibility of phase 2 reentry. Examples of (**A**) VF2 and (**B**) VF6 in the right ventricular preparation number 3. APD_80_ = action potential duration at 80% of repolarization; ECG = electrocardiogram; PVC = premature ventricular complex; RV ant. = anterior right ventricle; RV post. = posterior right ventricle; RVFW = right ventricular free-wall; RVOT = right ventricular outflow tract.

Taking measurements during the activity preceding the onset of VF, the local gradients of APD at 80% of repolarization (APD_80_) and RT at trigger origin sites (n = 16) were then compared with those at nontrigger sites (average of 3 pixel measurements taken in the nonaffected sites/normal areas for each preparation; N = 4). Figure 6 shows that VF triggers originated from sites harboring local APD_80_ and RT gradients significantly steeper than those found at nontrigger sites (15.9 and 15.8 ms/mm vs 1.5 and 3.0 ms/mm; *P* = .0004; up to 26.1 and 26.9 ms/mm vs 2.0 and 4.3 ms/mm). Two examples of optical traces are shown in Figure 7, in which the triggers emerge in the short APD area whereas the neighboring (distance of 5 mm) area of long APD is not yet repolarized. Trigger emergence could potentially be due to reexcitation of the short APD areas by the depolarized tissue in the long APD areas.

### Location of the initial VF drivers of sustained VF

**Figure 8:**
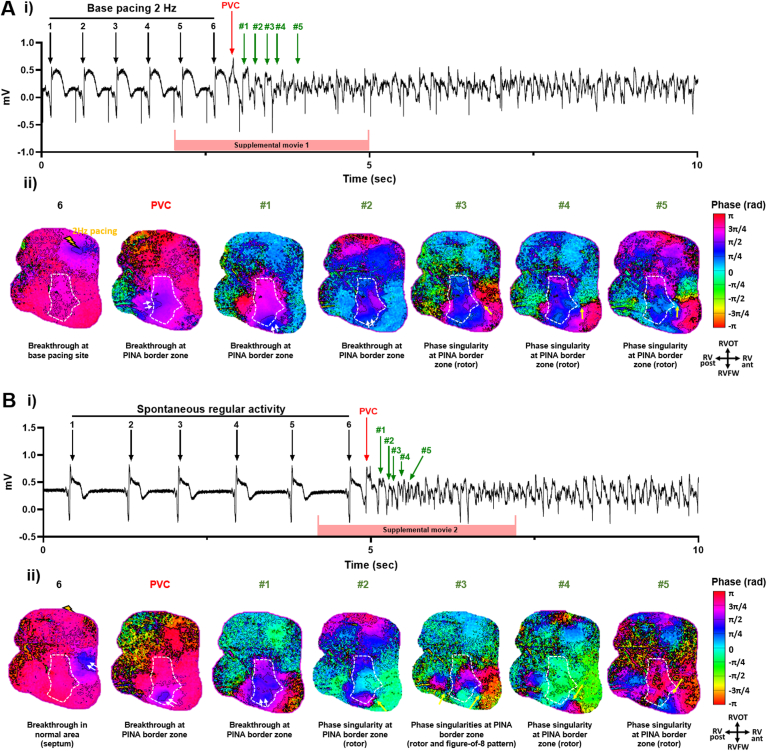
The initial drivers following ventricular fibrillation (VF) triggers are located in repolarization heterogeneity area. Examples of (**A**) VF2 and (**B**) VF6 onsets in the right ventricle number 3. (**i**) Pseudo-electrocardiograms (ECGs) and (**ii**) corresponding representative snapshots of the phase mapping movie showing the complex preceding the trigger, the trigger (premature ventricular complex [PVC]), and the first seconds after VF trigger, with 5 snapshots showing either repeated breakthroughs (*double white arrows*) at the site of origin of the initial trigger (border zone with steep repolarization time [RT] gradients) or singularity phases (*yellow arrows*) demonstrating transitions to rotors and figure-of-8 patterns. The temporal correspondence of Online Supplemental Movies 1 and 2 to the ECG is shown here. PINA = pinacidil; RV ant. = anterior right ventricle; RV post. = posterior right ventricle; RVFW = right ventricular free-wall; RVOT = right ventricular outflow tract.

Phase mapping during the few VF cycles following the trigger indicated a repetition of breakthroughs at sites contiguous to those of the initial trigger, at the border zone, with a transition to rotors as early as the second VF cycle. Two examples are presented in Figure 8 and Online Supplemental Movies 1 and 2.

## Discussion

This study shows the spontaneous initiation of VF using high-resolution mapping in a model of pharmacologically induced heterogeneous repolarization. It shows that triggers originate from the border zone of areas expressing a high dispersion/gradient of repolarization.

### Relevance of the tissue model

This study complements previously reported experimental models of localized repolarization heterogeneity by using intra-arterial infusion of pharmacological agents affecting the repolarization.^20^, ^21^, ^22^, ^23^, ^24^, ^25^, ^26^, ^27^, ^28^

We have used a single drug, pinacidil, a adenosine triphosphate-sensitive potassium channel activator, to minimize drug interactions at a dose of 20 μM (effective concentration giving 50% of the maximum effect on APD_80_ in our experimental conditions). Pinacidil was infused in a terminal branch of the right coronary artery to create a *localized* area of short APD (early RT), and this model was distinct from previous investigations in wedge or full organ preparations. In recent studies by Coronel et al^21^ and Cluitmans et al,^24^ 2 drugs (sotalol, or dofetilide, and pinacidil) were infused in different coronary arteries to obtain marked repolarization shortening; however, no spontaneous VF onset was obtained despite the marked repolarization gradient and the vulnerability to VF was investigated using an extrastimulation pacing protocol. In our study, we observed a high incidence of spontaneous VF initiations, and this high incidence was likely explained by differences in the protocol: a different area (right ventricle vs left ventricle) and topology of the heterogeneity created (more localized), and pacing in the right atria for them while pacing in the ventricle, so closer to the substrate, for us. Our experiments also require an electromechanical uncoupling, and we use a different perfusate (sodium bicarbonate vs blood) that provides a slightly higher level of extracellular K^+^ than blood, which could increase the resting membrane potential of cells and promote the appearance of PVCs. The high spatial resolution of optical mapping (0.39 mm) allowed us to identify the spatial emergence of spontaneous VF triggers and its specificities with regard to the underlying repolarization properties. Our model is therefore an excellent tool to study the trigger-substrate relation in the context of spontaneous arrhythmias.

### Spatiotemporal characteristics of the spontaneous VF trigger

The use of pseudo-ECGs showed that the spontaneous ectopies triggering VFs had shorter coupling intervals (mostly R-on-T) than PVCs not triggering VF, in line with previous clinical and experimental studies. However, this pattern was not fully sensitive (VF could start with long coupling) or specific (short-coupled PVCs not inducing VF), as other critical components are involved in VF initiation that are not visible on the ECG and because the mechanism of the first triggering complex and subsequent ones (reentry) are likely distinct. Previously, the heterogeneity and restitution properties of repolarization, the timing and location of trigger PVC, and the tissue conduction patterns have all been shown to interact in the context of repolarization dispersion.^20^^,^^21^^,^^24^ The activation sequence may indeed influence the local RT gradient by exacerbating the dispersion of repolarization induced by APD gradients. For example, an activation originating from the short APD area exacerbates the local RT gradients because of early AT (RT = AT + APD) compared with a ventricular activation sequence originating from a long APD area, which favors the formation of conduction blocks. This may explain why PVCs originating from the short APD area are more likely to degenerate into arrhythmias.

Our results indicated a close spatial relationship between triggers and the underlying repolarization “substrate,” a result also reported in various other arrhythmic scenarios.^9^^,^^10^^,^^13^^,^^15^, ^16^, ^17^^,^^24^^,^^26^, ^27^, ^28^, ^29^ Previous studies by Rivaud et al^30^ and Cluitmans et al^24^ have shown the importance of a steep repolarization gradient in promoting VF using pacing from the region of early repolarization. The steeper repolarization gradient was associated with a greater vulnerability to arrhythmia. Here, our study provides the first experimental evidence that, in our model of a localized repolarization abnormality in a perfused right ventricle, spontaneous PVCs inducing VF appear at the border zone of the early repolarization region at sites with large repolarization gradients. Our model based on a localized region of early repolarization is likely relevant to the broader group of repolarization syndromes, which have been investigated in other experimental models.^6^, ^7^, ^8^, ^9^, ^10^, ^11^^,^^23^^,^^24^^,^^30^

We observed through optical mapping that the ventricular area which had repolarized early (ie, recovered excitability) was consistently the origin of spontaneous triggers. This colocation of triggers within the area of maximal repolarization dispersion is unlikely coincidental and suggests a direct mechanistic link between the substrate and the trigger, as previously described as R-from-T mechanisms by Qu and coworkers.^31^^,^^32^ In this concept, the PVC is no longer considered to be a substrate-independent event but rather generated by the repolarization pattern of the preceding beat, which is precisely spatially heterogeneous in this substrate area.^31^^,^^32^ The first experimental evidence of R-from-T mechanisms was the so-called phase 2 reentry, initially described by Di Diego and Antzelevitch,^27^ where the electrotonic influence between neighboring sites leads to propagation of maintained action potentials (late repolarization) to contiguous early repolarization sites, resulting in local tissue reexcitation. This is a tissue-level (not cell-level) mechanism, which nevertheless requires strong RT gradients to be effective. The reported effectiveness of pinacidil in suppressing rather than favoring arrhythmic activity caused by triggered activity or abnormal automaticity at the cellular level also supports this hypothesis.^33^^,^^34^

However, this phase 2 reentry phenomenon cannot explain the emergence of the longer-coupled triggers of sustained VFs found in our study (38% of the R-off-T wave). The injury current mechanism described in the context of acute ischemia^35^ could explain the emergence of PVCs at the periphery of the pinacidil-perfused area. Indeed, the pinacidil-induced accumulation of extracellular potassium could increase the resting membrane potential of the cells in this area, causing an electrotonic current flowing to the adjacent normal area during the diastole, and a depolarization at the interface between normal and pinacidil-perfused tissue (PVC) degenerating into reentry when it reaches the nonrefractory early RT area. The timing of this scenario may allow an R-off-T wave to induce reentry and arrhythmia; however, further investigations are needed to better understand the mechanisms involved in these specific R-off-T cases.

### Spatial correlation between VF drivers and the repolarization substrate

In line with our previous clinical study,^36^ we demonstrated that in our model the initial VF drivers following the spontaneous trigger of sustained VF are colocalized with the substrate at the border zone of repolarization dispersion. Unfortunately, as our experiments do not allow us to visualize the 3-dimensional propagation patterns, we cannot identify the precise mechanisms by which the initial PVC progresses in reentry. Qu et al^31^^(p7)^ hypothesized that “because phase 2 reentry is due to an ongoing dynamic tissue instability, once the first episode of phase 2 reentry occurs, the repolarization heterogeneities become very dynamic, resulting in additional phase 2 reentry events perpetuating the arrhythmia.” This could explain the recurrent breakthroughs in the initial site of PVC origin (initial phase of focal arrhythmia) if they are indeed caused by phase 2 reentry. However, according to these authors, PVCs triggered by an R-from-T mechanism (eg, phase 2 reentry) are supposed to propagate spontaneously in a unidirectional fashion,^32^ allowing the formation of reentries. In our case, PVCs on the contrary propagate in all directions, leaving open the question of the pathway by which focal arrhythmias degenerate into reentrant arrhythmias.

### Study limitations

This work is subject to some limitations: (1) the model used is mainly relevant to cardiac disorders involving repolarization abnormalities (such as long, short, and early repolarization). (2) The use of pinacidil as a pharmacological tool to create APD shortening could represent a limitation due to previously reported potential impact on arrhythmogenicity; however, it has also been shown that pinacidil prevents arrhythmogenicity.^33^^,^^34^ (3) The need for continuous illumination of the tissue to record random VF triggering was associated with a progressively lower amplitude of the optical signals and hence a lower signal-to-noise ratio. While trigger mapping was clear as it abruptly deviated from a slow rhythm, the optical signals beyond the initial few 4–5 complexes must be interpreted with caution. It also affected our ability to map VF continuity in these experiments. (4) Although the heterogeneities created were present in both the epicardium and the endocardium, only the epicardial side was analyzed with regard to previous observations where maximal heterogeneity is found in the epicardium. (5) The experiments were performed in ex vivo conditions, so the results have to be carefully considered; in vivo and chronic factors can play a role, especially in J-wave syndromes, including sympathovagal balance, body temperature, and metabolic changes. (6) The inherent differences between the pig and human right ventricles, specifically the lack of transient outward current, which plays a critical role in facilitating the development of repolarization heterogeneities under various conditions, including exposure to pinacidil, may limit the clinical relevance of our findings to human physiopathology.

### Clinical implications

The spontaneous VF triggers and the vulnerable substrate of repolarization heterogeneity can be spatially and causally associated and should not be considered separately in VF therapies. The arrhythmogenic PVC triggers are spatially correlated with the repolarization substrate and should be considered as an emerging target to treat recurrent VFs in the subgroups of patients presenting PVCs in both electrophysiologically and structurally impaired areas. Our results further suggest that substrate-based ablation may, in some cases, be sufficient to eliminate triggers as well, even when those that cannot be mapped per procedure. Our findings on the proximity of triggers and substrate could also be considered in the risk stratification of life-threatening arrhythmias.

## Conclusion

The presented model of localized repolarization heterogeneity is able to consistently produce spontaneous VF initiation. It demonstrates that VF triggers originate from the border zone of repolarization dispersion at sites of steep repolarization gradients.

## Disclosures

The authors have no conflict of interest to declare.

## Appendix Supplementary Data

Supplemental Movie 2

SupplementalMaterial
