# Supplementary material for: Origin of ventricular fibrillation triggers in a model of localized repolarization heterogeneity
Source: Heart Rhythm. 2025 May;22(5):1345–54. doi: 10.1016/j.hrthm.2024.10.027 (PMC12048301; doi:10.1016/j.hrthm.2024.10.027)
Supplement: SupplementalMaterial [file mmc3.docx]

Supplementary Materials for

**Origin of ventricular fibrillation triggers in a model of localized repolarization heterogeneity**

Estelle Renard^[[1]](#footnote-1)*^, PhD, Michel Haïssaguerre^1,^^[[2]](#footnote-2)^, MD, Laura R Bear^1^, PhD Olivier Bernus^1^, PhD

# Supplemental Methods

This text provides additional information about methods mentioned in the main manuscript. The workflow of the experiments is summarized in the Supplemental Figure 1.

## Animals

All the protocols used in this study were in accordance with the European Parliament Directive 2010/63/EU on the use of animals for scientific purposes. Protocols were approved by the local ethics committee of the University of Bordeaux CEEA-050 and the French government (approval n° A33-318-3). All efforts were made to avoid animal suffering and stress.

The experiments were performed on RV preparations from “Large White” male pigs aged 3 months ± 2 weeks and weighing 40.1 ± 1.9 kg. The animals arrived at the laboratory at least one week before the experiment. They were housed in stalls in groups of 2 to 3 according to arrival, at a temperature of 20.4 ± 1°C and a humidity of 30%, in a 12-hour light/dark cycle with free access to food (pellets or SENIOR UNIC meal) and drinking water.

## Anesthesia and cardiac removal

After a 12-hour fasting period, the animals were premedicated by intramuscular injection of a mixture of ketamine (10-20 mg/kg, Vibrac), acepromazine (0. 1 mg/kg, Vetoquinol) and buprenorphine (0.09 mg/10kg, Axience), allowing them to be transferred to the operating room. An intravenous perfusion line was placed through which a bolus of Propofol (1mg/kg, Braun and Mylan) was injected to induce deep anaesthesia. An orotracheal intubation was immediately set up to ventilate the animals at a rate of 10 to 15 movements per minute (10 ml/kg, Hallowell 2000, TEM-SEGMA) with a mixture of air (50% O_2_ / 50% CO_2_) and isoflurane (1.5 to 3%) allowing the maintenance of deep anaesthesia until sacrifice. After performing a total sternotomy, the rib cage was opened to expose the heart. Checks for the absence of lung stains and pericardial adhesions were performed to assess their cardiorespiratory health status. To prevent blood clotting during removal, heparin was injected intravenously (200 IU/kg, PanPharma) and the pericardium was opened before the pig was euthanized *via* intravenous injection of a lethal dose of sodium pentobarbital (80 mg/kg, Axience and Sanofi). Rapidly, the decline in cardiac activity was noted, allowing excision of the still beating heart which was immediately placed in a cold cardioplegia solution (0-4°C) composed of (in mM): 110 NaCl; 1.2 CaCl2; 16 KCl; 16 MgCl2; 10 NaHCO3; 9 glucose, and supplemented with heparin (200 IU/L). The aorta was rapidly cannulated to retroperfuse the heart with the same solution, simultaneously removing all blood from the chambers and coronary circulation and stopping the heartbeat due to excessive potassium, thus making the tissue inexcitable and considerably reducing its oxygen consumption. This step is crucial to avoid ischemia and to maintain the integrity of the tissue throughout the dissection and preparation of the RV.

## Dissection and preparation of the right ventricle

The explanted and rinsed heart was then placed in a dissection dish on ice containing cold cardioplegia solution throughout the dissection. First, the left atria was opened to provide access to the left ventricular cavity, allowing an incision to be made along the left anterior descending artery (LAD) on the anterior left ventricle's side from the base to the apex. Then, passing through the superior cava vein, the posterior RV was cut along the posterior interventricular septum to the apex, before separating the two ventricles by incising the septum from the apex towards the base. The aorta was then opened and the ostia of the right coronary artery (RCA) and left coronary artery (divided into LAD and circumflex artery) were isolated, allowing to open the pulmonary artery and revealing the RVOT. The preparation was more precisely cut by removing unnecessary parts (cava veins, left atria, non-perfused part of the posterior RV, interventricular septum) as well as the moderator band which prevents the total opening of the preparation. Both LAD and RCA arteries were then cannulated and perfused with cold cardioplegic solution while the coronary artery leaks opened during dissection were closed. Large diameter arteries were ligated with a clamp and braided sutures while smaller leaks required the use of an electric scalpel to cauterize the arteries. This step of closing the coronary system increases the perfusion pressure and thus allows homogeneous perfusion of the entire RV. The venous system must be left open to allow drainage of the perfusate and to avoid oedema during the experiments. The preparation was then fixed onto a frame with reasonable tension on the tissue to keep the epi- and endocardial surfaces of the myocardium fixed and as flat as possible (relative to the mapping focal plane). The final RV preparations stretched over the frame measured approximately 10 x 8 cm and allowed optical and electrical measurements to be made on the RVOT and RVFW perfused by the LAD and RCA which have their respective perfusion territories.

## Intracoronary perfusion system

The RV preparations were perfused in an intra-coronary perfusion system inspired by the Langendorff model: each of the two previously cannulated coronaries was perfused with physiological sodium bicarbonate (Tyrode) solution (in mM: NaCl 130; NaHCO3 24; NaH2PO4 1.2; MgCl2 1; KCl 4; CaCl2 1.8; glucose 5.6) at a constant flow rate of 25 mL/min controlled by peristaltic pumps (Minipuls® 3, Gilson). The solution was heated to 38 ± 0.5 °C by a water bath circuit and its pH was maintained at 7.4 by oxygenation with a mixture of oxygen and CO_2_ (95% O_2_, 5% CO_2_). The RV were also immersed in a transparent plexiglass tank whose solution content was continuously renewed and maintained at 38 ± 0.5 °C by the implementation of a closed superfusion circuit (perfusate recycling). As this system reproduces conditions close to *in vivo* physiology, the immediate consequence of perfusing the ventricular preparations was the recovery of cardiac contractions.

The preparations were paced at different frequencies at two sites on their epicardial surface using bipolar tungsten electrodes (World Precision Instruments) connected to a constant current pacemaker (DS3 Isolated Current Stimulator, Digitimer Ltd) which was controlled using LabChart software (AD-Instruments) and a PowerLab system (AD-Instruments). The overall electrical activity of the heart was also monitored using an amplified pseudo-ECG system (Animal Bio Amp and PowerLab, AD Instruments) which recorded activity between a positive and a negative electrode affixed to each side of the preparation in a reproducible manner, and a reference electrode immersed in solution.

Creation of a localized repolarization heterogeneity

The first part of each protocol consisted of recording the basal properties of the RV corresponding to the control condition (Ctrl), i.e. the basal Tyrode perfusion and superfusion circulation as previously described. These basal conditions were subsequently modified to create a localized repolarization heterogeneities in an area representing about 5-10% of porcine right ventricles. This experimental model was created by locally perfusing a K-ATP channels opener, the pinacidil (PINA; 20µM; N=16) *via* an epicardial catheter inserted in a terminal branch of the RCA.

In each preparation, optical mapping and ECG measurements were made under Ctrl conditions, after the insertion of the catheter, which infused only Tyrode, and after the creation of heterogeneities by pinacidil perfusion through the catheter.

## Ex vivo optical mapping

-Decoupling of the excitation-contraction process and cardiac electrical activity probing: Each experiment started by waiting for the ventricular preparation to stabilize under pacing for 15 to 20 minutes. In order to perform subsequent optical mapping recordings, it was necessary to inhibit cardiac contractions by adding blebbistatin (Enzo Life Sciences) to the recirculating perfusate to obtain a final concentration of 7.5 µM; additional boluses of blebbistatin (50 mL at 60 µM in each cannula) could be reasonably added if beating reoccurred. Once RV contractions were abolished, a voltage-sensitive potentiometric dye (di-4-ANEPPS, Biotium) was bolused into both coronaries (10 mL bolus at 20 µM in each) to record optical action potentials. Identical boluses with half concentration were added when the recorded optical signals were no longer of sufficient intensity. These probe was excited at 530 nm by uniform illumination of the EPI and ENDO surfaces by four pairs of monochromatic light emitting diodes (LEDs) (OptoLED, Cairn Research). The fluorescence emitted by the dye was filtered through bandpass filters of 650 ± 50 nm and was recorded by two cameras located on either side of the ventricular preparation and connected to a high-performance acquisition system. The cameras was the MiCAM02-CMOS for the 13 first right ventricles of this study, they were then replaced by the MiCAM03-N256 (SciMedia, Brain Vision) for the 3 last experiments to enable continuous optical recording to facilitate the optical mapping of spontaneous VF onsets which, by definition, can occur at any time. The 100 x 100 pixels (MiCAM02-CMOS) and the 256x256 pixels (MiCAM03-N256) optical images from the EPI and ENDO had a respective spatial resolution of 0.9 mm and 0.39 mm and a temporal resolution of 1 kHz. The optical signals were digitized and visualized on the computer during the experiment using the specialized software BrainVision Analyse (Sci Media BrainVision) for the first system, and BV WorkBench (Sci Media BrainVision) for the second.

-Stimulation protocols: The primary objective of each of the experiments was to provide proof of concept i.e. to validate the creation of the expected kind of substrate, therefore the electrophysiological properties were first studied following bipolar stimulations (2 ms pulses) applied at two epicardial stimulation sites, one involving propagation of the impulse in the physiological direction from the apex of the ventricles towards the base (Site 1: Apex), the other involving retrograde propagation of this impulse (Site 2: Base). It should also be noted that the Apex site was always located on the lateral side, in the perfusion territory of the RCA, while the BASE_EPI_ site was in the anterior part perfused by the LAD. Restitution protocols consisting of increasing the stimulation frequency from 1 to 4 Hz in 0.5 Hz increments were performed for each stimulation site. During these protocols, optical acquisitions were performed by sequentially illuminating the EPI and ENDO surfaces because transillumination induced contamination of the signals obtained on each side by those of the opposite side (due to a very thin wall thickness of the RV).

Subsequently, and after a recovery time (5 min), the spontaneous activity was optically recorded during 60 seconds without pacing. These protocols were reproduced identically for each of the conditions of each experiment.

During the local perfusion of pinacidil creating the localized heterogeneity of repolarization, the aim was to record the spontaneous VF onsets. With the MiCAM02-CMOS cameras, it was a matter of luck to get a recording at the right moment, as acquisition times were limited to 8 seconds. That is why the new system with the MiCAM03-N256 cameras was acquired allowing quasi-continuous optical recordings thus facilitating optical mapping of spontaneous VF onsets.

-Optical signals and ECG analysis: Pseudo ECGs were recorded to quantify spontaneous arrhythmogenicity and analyze whether each VF onset was preceded by a PVC in the 16 right ventricles (N=16; LabChart® acquisition system and software, AD Instruments). .

The optical signals were analyzed using the software BV Workbench (Sci Media, Brain Vision). The signal was filtered (120 Hz; 3 mm spatial filter; 3 ms temporal filter) and normalized, followed by averaging all the optical AP contained in each acquisition. First, global measurements of the different parameters were performed on each EPI and ENDO surface by cropping the optical images to exclude areas where the signal was distorted and the signal-to-noise ratio was too low to avoid biasing the results. The EPI and ENDO masks were specific to each of the ventricular preparations but were selected in the most reproducible way possible between each experiment. In a second step, parameters were studied locally to compare the properties of two regions of interest (ROIs) of 10 pixels by 10 pixels selected in mirror on the EPI and ENDO images for each experiment: an unmodified control area (named "Norm.") compared to an area either perfused by pinacidil (named "PINA"). The action potential at 80% of repolarization (APD_80_) and activation time (AT) values for each pixel of the epi- and endocardial surfaces were calculated allowing to generate APD and AT maps (with an interval of 2 ms chosen for the isochrones connecting pixels with the same AT). Optical AT were defined by the time to reach the maximal derivative of the AP upstroke after a stimulation and APD_80_ were determined as the time difference between AT and 80 % of repolarization. Mean epicardial conduction velocities were measured by calculating the gradient of the activation maps in the ROIs. Surface gradients of APD_80_ (APD_80_ gradient) and CV (mean CV gradient) representing the difference in values between the two local ROIs on each side reported at the distance between these two ROIs were also determined. Repolarization time (RT) of each pixel was considered as the time required for the AP to reach 80% of its repolarization after a stimulation, i.e., RT = AT + APD_80_. The RT dispersion was considered to be the time difference between the minimum RT and the maximum RT of a given surface.These above analyses allowed to confirm the creation of the substrate of repolarization heterogeneity without affecting the conduction.

For the analysis of arrhythmias, spontaneous events (sustained arrhythmias occurring independently of any voluntary induction protocol and during continuous pacing < 2 Hz) were quantified. The optically mapped VFs onset (n=16/N=4) were analysed by phase mapping allowing to observe the origin site of each focal activity including those of triggering-PVCs.

## Statistical analysis

Statistical tests were carried out using Prism (GraphPad Software). The data were expressed as mean ± standard deviation (std). The mean comparison of two paired or unpaired variables was performed using the appropriate Student statistical test (paired t-test or unpaired t-test) or its non-parametric equivalent (respectively Wilcoxon signed rank test or Wilcoxon rank sum test) when the populations did not have a normal distribution (tested by Shapiro-Wilk). The comparison of multiple variables was carried out using non-parametric equivalent of the ordinary one-way (unpaired) ANOVA analysis (Kruskal-Wallis test) implemented by Holm-Sidak multiple comparison tests. Fisher's exact tests were used to test the independence of two variables when required.

Differences or dependences were considered statistically significant if the p-value was <0.05.


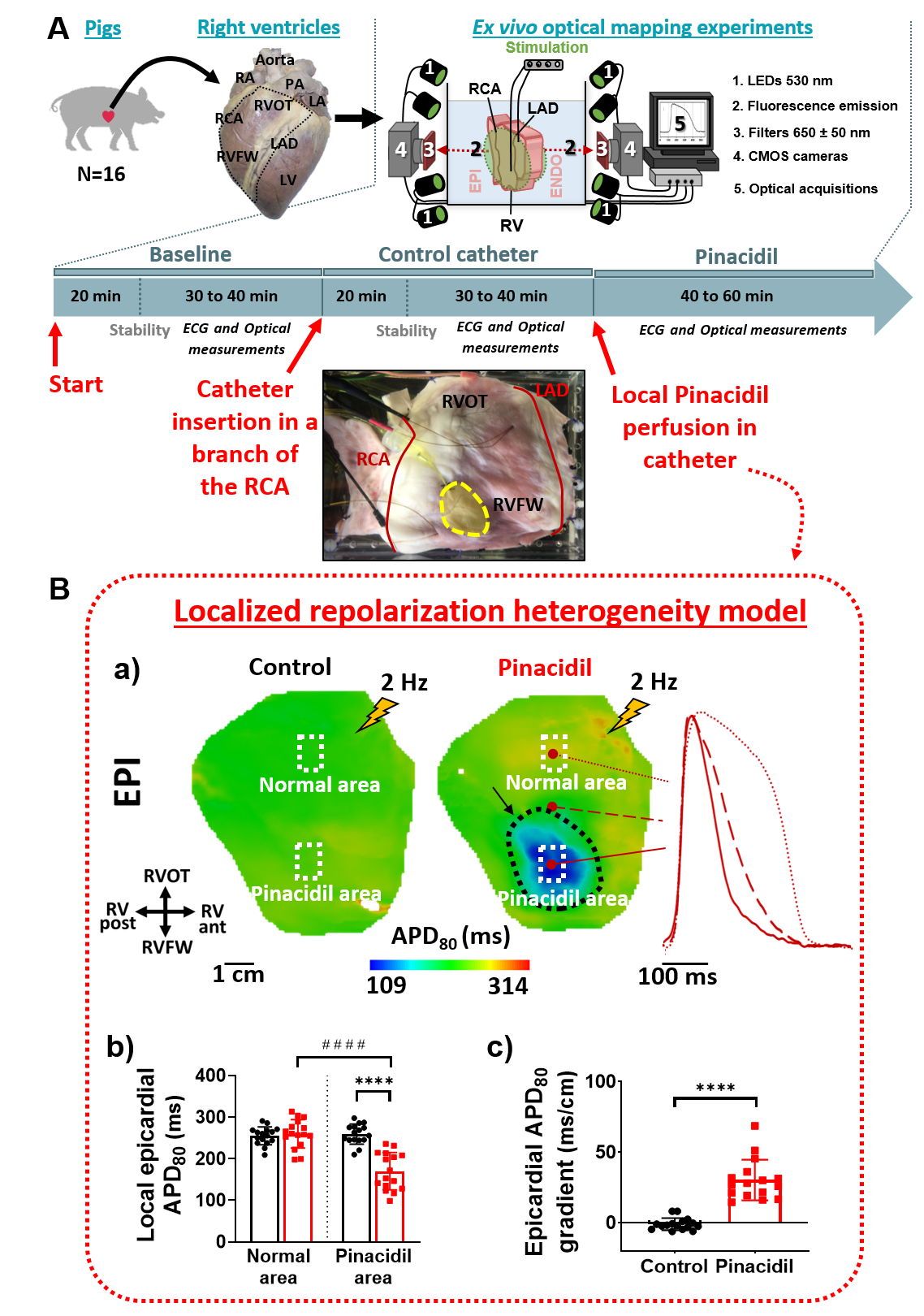


# Supplemental Figure 1

**Workflow of the experiments**

**(A)** The right ventricles from pig’s hearts were dissected and perfused in a Langendorff-like system by canulating the left anterior descending (LAD) and the right coronary arteries (RCA). Optical mapping measurements as well as pseudos-ECG recordings were done in baseline, after a catheter insertion in a terminal branch of the right coronary artery (Control Catheter), and during the local infusion of the K-ATP channels opener, Pinacidil (20 µM) through this catheter (Pinacidil). **(B)** This allowed the creation of our model of localized repolarization heterogeneity because of the local shortening of action potential duration (APD80) in the « pinacidil area » delimited by dotted line. **a)** Representative maps of action potential duration (APD_80_) and optical action potential traces before and after local pinacidil perfusion (black arrow: position of the catheter for pinacidil perfusion in the dashed lines area; white dotted squares: 10x10 pixels normal and pinacidil regions of interest). Effect of pinacidil perfusion on the **b)** repolarization (APD_80_) parameters in normal vs. pinacidil areas, and **c)** their relative surface gradients.

N=16 – (B.b) *: RM one-way ANOVA for Pinacidil vs. Control comparisons; #: Ordinary one-way ANOVA for Control (Normal area) vs. Control (Pinacidil area) and Pinacidil (Normal area) vs. Pinacidil (Pinacidil area). (B.c) Wilcoxon signed rank test (non-parametric paired t-test); 4 symbols indicates p<0.0001


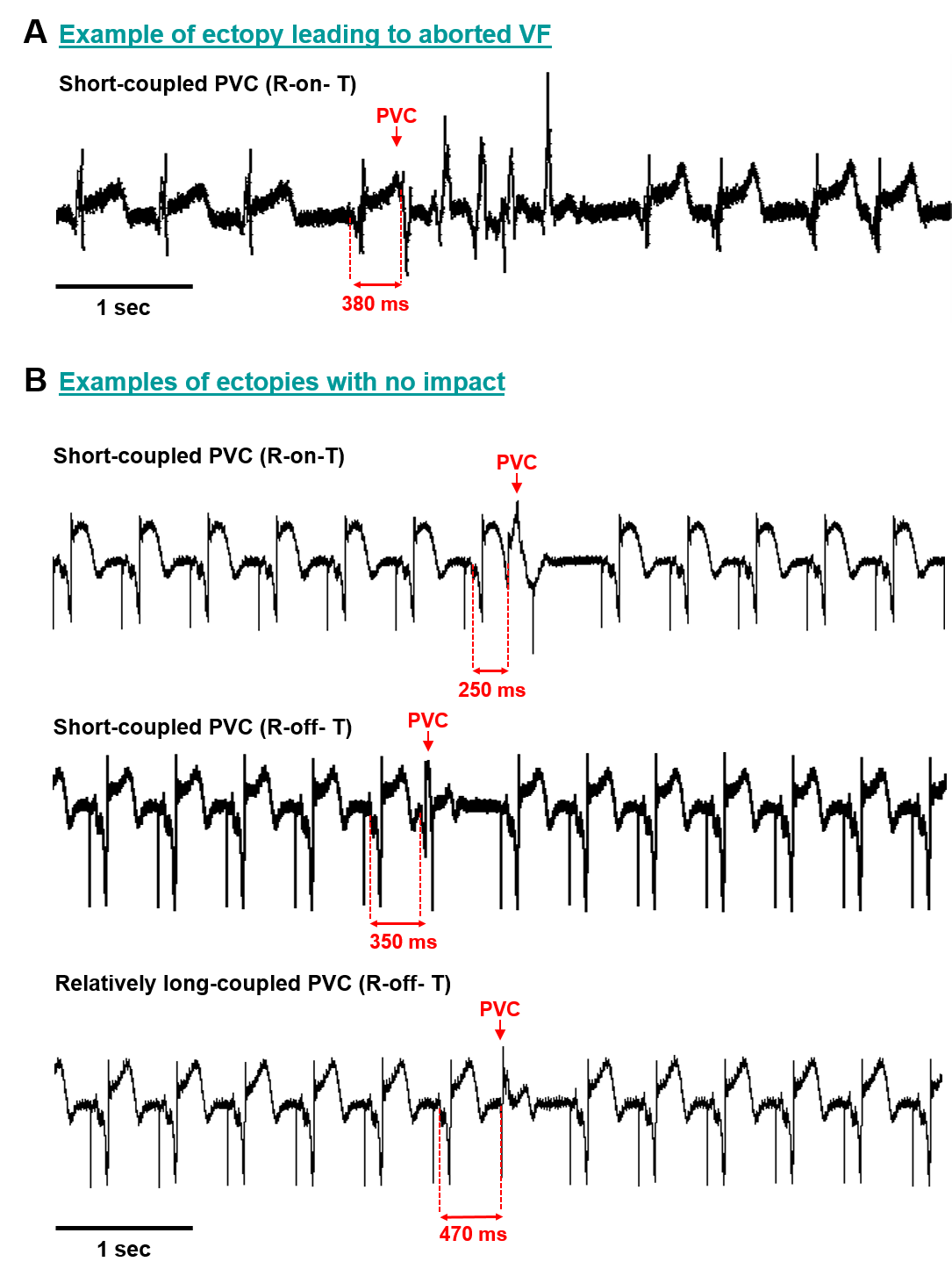


# **Supplemental Figure 2**

**ECG recordings showing examples of PVCs with distinct consequences**

**(A)** PVC leading to quicly aborted VF. **(B)** PVCs with different coupling interval which had no impact.

# **Supplemental Movie 1**

**Phase mapping movie of VF2 onset in right ventricle no.3**

The phase mapping of the VF2 onset in right ventricle n°3 shows, during the first seconds after VF trigger (PVC), repeated breakthroughs at the site of origin of the initial trigger, at the border zone, as well as transitions to rotors located in this same zone. The temporal correspondence of the movie to the ECG is shown in panel A.i of Figure 8.

# **Supplemental Movie 2**

**Phase mapping movie of VF6 onset in right ventricle no.3**

The phase mapping of the VF6 onset in right ventricle n°3 shows, during the first seconds after VF trigger (PVC), repeated breakthroughs at the site of origin of the initial trigger, at the border zone, as well as transitions to rotors and figure-of-eight patterns in this same zone. . The temporal correspondence of the movie to the ECG is shown in panel B.i of Figure 8.

1. *Univ. Bordeaux, INSERM, CRCTB, U 1045, IHU Liryc, F-33000 Bordeaux, France*

   [↑](#footnote-ref-1)
2. CHU de Bordeaux, Service *de Cardiologie-électrophysiologie et stimulation cardiaque Hôpital Haut Lévêque*, INSERM, U 1045, F-33000 Bordeaux, France

   *Correspondence to:

   Estelle RENARD

   estelle.renard@ihu-liryc.fr

   IHU Liryc, Avenue du Haut Lévêque, 33600 Pessac, France

   **This files includes :**

   Supplemental Methods

   Supplemental Figures 1 to 2

   Supplemental Movies 1 to 2 [↑](#footnote-ref-2)
